# Patterns of Long Term Care in 29 European countries: evidence from an exploratory study

**DOI:** 10.1186/1472-6963-11-316

**Published:** 2011-11-18

**Authors:** Gianfranco Damiani, Valentina Farelli, Angela Anselmi, Lorella Sicuro, Alessandro Solipaca, Alessandra Burgio, Domenica Fioredistella Iezzi, Walter Ricciardi

**Affiliations:** 1 Department of Public Health, Università Cattolica del Sacro Cuore - Rome, Italy; 2 Health and Assistance Unit, Italian National Institute of Statistics, ISTAT - Rome, Italy; 3 Social Statistics, Università di Tor Vergata - Rome, Italy

## Abstract

**Background:**

The challenges posed by the rapidly ageing population, and the increased preponderance of disabled people in this group, coupled with the rising level of public expenditure required to service the complex organization of long term care (LTC) delivery are causing increased pressure on LTC systems in Europe. A pan-European survey was carried out to evaluate whether patterns of LTC can be identified across Europe and what are the trends of the countries along them.

**Methods:**

An ecological study was conducted on the 27 EU Member States plus Norway and Iceland, referring to the period 2003-2007. Several variables related to organizational features, elderly needs and expenditure were drawn from OECD Health Data and the Eurostat Statistics database and combined using Multiple Factor Analysis (MFA).

**Results:**

Two global Principal Components were taken into consideration given that their expressed total variance was greater than 60%. They were interpreted according to the higher (more than 0.5) positive or negative correlation coefficients between them and the original variables; thus patterns of LTC were identified. High alignment between old age related expenditure and elderly needs characterizes Nordic and Western European countries, the former also having a higher level of formal care than the latter. Mediterranean as well as Central and South Eastern European countries show lower alignment between old age related expenditure and elderly needs, coupled with a level of provision of formal care that is around or slightly above the average European level. In the dynamic comparison, linear, stable or unclear trends were shown for the studied countries.

**Conclusions:**

The analysis carried out is an explorative and descriptive study, which is an attempt to reveal patterns and trends of LTC in Europe, allowing comparisons between countries. It also stimulates further researches with lower aggregated data useful to gain meaningful policy-making evidence.

Please see related article: http://www.biomedcentral.com/1741-7015/9/124

## Background

Demographic trends, financial issues and organizational features are the factors that mainly affect current policies of long term care (LTC) in Europe. The OECD defines LTC as "a range of services needed for persons who are dependent on help with basic activities of daily living over an extended period of time"[1].

The irreversible process of population ageing is mostly due to the low fertility rate, the increasing life expectancy, both at birth and at age 65, the aging of the baby boom generation (those born soon after the Second World War, who are now progressing towards retirement age), and the uncertain effects of international migration inflows [2].

In 2008, the number of persons aged 65 and over, representing 17% of the total population, surpassed the number of children (aged below 15 years). According to the projections, the number of elderly will almost double in the near future, rising from 85 million in 2008 to 151 million in 2060. The number of the oldest old (aged 80 and over) is projected to increase more rapidly, almost tripling from 22 million in 2008 to 61 million in 2060 [3].

This demographic trend will lead to new patterns of growing morbidity among the elderly. This means an increase in degenerative and chronic diseases, often associated with functional restrictions and disability. This situation is generally related to limitations and dependency on help for one or more of the basic activities of daily living (ADLs), such as eating, washing/bathing, dressing, getting in and out of bed and any other clearly defined self-care activity [1,3,4].

Trends for disability are not always clear. In 2007 a study reported non-uniform past trends in disability among some European elderly populations. Denmark, Finland, Italy and the Netherlands showed a falling prevalence of disability, Belgium and Sweden were characterized by a rising trend, while for the United Kingdom and France it was not possible to draw any definitive conclusion because different sources provided diverging results [4]. Additionally, the future prevalence of disability is difficult to predict because it is not clear to what extent the increased longevity will be characterized by additional life years spent in good health (disability-free life expectancy) [4,5]. However, recent OECD forecasts show an overall upward trend in the share of disabled elderly who will be in need of assistance and will consequently sustain demand for LTC [4]. This will result, *inter alia*, in a growth of public expenditure on the elderly population.

Public expenditure on LTC varies widely across Europe, ranging from 0.2% of GDP in the Czech Republic and Portugal to more than 3% in Sweden and the Netherlands. According to OECD predictions, it is expected to increase by 1.2% of GDP on average between 2005 and 2050 [6]. In such a scenario, characterized by an increasing share of disabled elderly and by rising expenditures, it is crucial for countries to reorganize their delivery systems, finding the balance between formal (more expensive) and informal (less expensive) care [6,7].

Regarding the expenditure, on the level of formally-provided LTC services a wide variability can be observed across countries. The provision of LTC beds in institutions (other than hospitals) ranges from less than 2% of the population aged 65 and over in Italy to 8% in Sweden, while the percentage of the elderly who are cared for either in institutions or at home ranges from less than 5% in Italy to more than 20% in Norway. In addition, it should be noticed that home care is everywhere much more developed than residential care, thus promoting the concept that the OECD has been calling for some years "ageing in place" [8].

Taking into account the demographic, financial and organizational factors considered so far, a study was carried out with the aim of performing a comparison across the European LTC systems. Our main research questions were: Can patterns of LTC be identified across Europe? What is the dynamic of the countries along these patterns?

## Methods

An ecological study was conducted on the 27 EU Member States plus Norway and Iceland, covering the years from 2003 to 2007. Official data provided by the OECD and Eurostat were used to calculate indicators [8,9]. The indicators included in the study were the following:

- The number of LTC beds in institutions (other than hospitals) per 100 population aged 65 and over,

- The number of LTC recipients in institutions (other than hospitals) per 100 population aged 65 and over,

- The number of LTC recipients at home per 100 population aged 65 and over,

- Share of people aged 80 and over per 100 total population,

- Proportion of subjects aged 65 and above who perceive themselves to have bad or very bad health,

- Proportion of subjects aged 65 and above who perceive themselves to have limitations in daily activities (activity restrictions for at least the past six months);

- Total LTC expenditure (HC.3 + HC.R.6) in million Euros per 100 population aged 65 and over,

- Social protection benefits for old age in million Euros per 100 population aged 65 and over

Short definitions and scientific sources of the variables are provided in Table 1; for a detailed description please see Additional file 1.

**Table 1:** Short definition and source of the variables

Variable	Definition	Source
LTC BEDS IN INSTITUTIONS	Beds in all types of nursing and residential care facilities dedicated to long-term nursing care and beds for palliative care in all types of nursing and residential care facilities	OECD Health Data 2009
LTC RECIPIENTS IN INSTITUTIONS	People receiving formal (paid) LTC in institutions (other than hospitals). The services received can be publicly or privately financed	OECD Health Data 2009
LTC RECIPIENTS AT HOME	People receiving formal (paid) LTC at home. The services can be publicly or privately financed	OECD Health Data 2009
SELF-PERCEIVED HEALTH AS BAD OR VERY BAD, PEOPLE AGED 65	Auto-evaluation of the general health state (i.e. any temporary health problem is not considered) by respondents	Eurostat Statistics
SELF- PERCEIVED LIMITATIONS IN DAILY ACTIVITIES, PEOPLE AGED 65+	Auto-evaluation by the respondents of the extent of which they are limited in activities people usually do because of health problems for at least the last six months	Eurostat Statistics
TOTAL LTC EXPENDITURE(HC.3+HC.R.6)	It includes "health" (HC.3) and "social" (HC.R.6) components of LTC. HC.3 refers to "Services of long term nursing care": it is the medical component of LTC. HC.R.6 refers to " Administration and provision of social services in kind to assist living with diseases and impairment"	Eurostat Statistics
SOCIAL PROTECTION BENEFITS OLD AGE	Benefits for the old age function include (1) cash benefits, such as old age pensions, anticipated old age pensions, partial retirement pension, care allowance and other cash benefits, and (2) benefits in kind, such as accommodation, assistance in carrying out daily tasks, other benefits in kind	Eurostat Statistics
POPULATION OVER 80	Population aged over 80 years	Eurostat Statistics

### Statistical analysis

Multiple Factor Analysis (MFA) was used to combine the available data [10]. MFA expands Principal Component Analysis (PCA) to the analysis of 3D structured data, where the same variables are measured on the same individuals at various times, forming a matrix X (with dimensions I * J* K) [11]. The variables *I *in the study were the indicators listed above, the individuals *J *were the countries and the groups *K *the five years from 2003 to 2007. Data in the matrix were standardized to overcome large differences in the range and units of the measured variables.

First of all, MFA performed separate PCAs on each year's elementary matrix (sub-matrix X_k_). Secondly, all the elementary matrices were normalized by dividing all their elements by the root of the first eigenvalue of their respective PCA. All these weighted variables made a general matrix and a global PCA was performed on it. It generated global Principal Components (PCs) or factors that were linear combination of the variables and maximized the variance among data [12-14].

PCs that accounted for at least a total variance of 60% and could be meaningfully interpreted were retained to explain enough variance with as few meaningful factors as possible [15,16].

They were interpreted according to the higher (>0.50) positive or negative correlation coefficient between them and the variables [15].

The global PCA finally constructed a factorial space, in which the global PCs were represented by axes and each country by five partial positions (one for each year) as well as by a gravity centre, representing the average spatial pattern for the country [17]. Combining the interpretation of the axes, four main patterns of LTC were identified. With respect to these patterns, a static comparison between EU countries was carried out considering their gravity centre points.

It was possible to make a dynamic comparison according to the trajectories of the countries defined on the factorial space, the trajectories being characterized by the shape and length of the arrows connecting the partial representations for each country. Linear arrows were interpreted as clear dynamic along the axes, circular lines as overall stable movement, and broken and irregular arrows were interpreted as unclear trends [18].

The French SPAD Package Software 5.0 was used to perform the analysis.

## Results

### Patterns

MFA generated five PCs. Two factors were retained which explained a total variance of 59.28%. (Table 2).

**Table 2:** Table of eigenvalues and variance in the global Principal Component Analysis

Global PCs	Eigenvalue	Variance (%)	Total variance (%)
1	4.7602	43.99	43.99
2	1.655	15.29	**59.28**
3	1.0773	9.96	69.24
4	0.8069	7.46	76.7
5	0.7016	6.48	83.18

**Figure 1:**
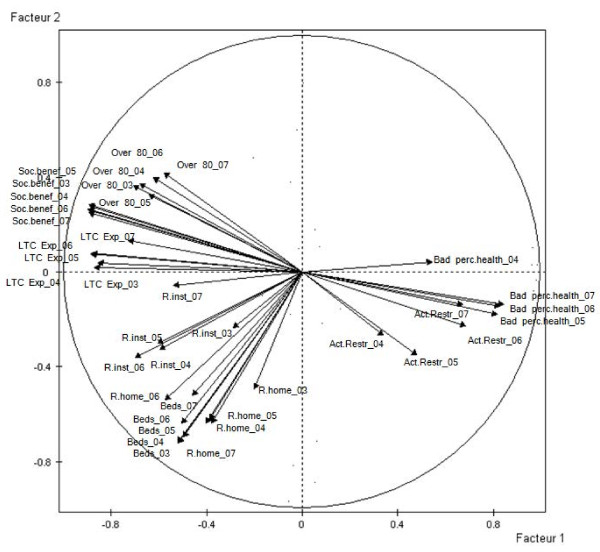
**Correlation circle of the variables on the two Principal Components (Factor 1 and Factor 2)**. Beds: beds in institutions; R. inst: recipients in institutions; R. home: recipients at home; Over 80: share of people aged 80 and over; Bad perc. health: self-perception of health as bad or very bad; Act. restr: self-perceived restrictions on activities; LTC Exp.: LTC total expenditure; Soc. benef.: social protection benefits for old age. Numbers represent the corresponding year for each variable.

The PCs were interpreted according to the correlation coefficients represented in the correlation circle (Figure 1). The first factor was always highly and negatively correlated with variables expressing the total LTC expenditure, the social protection benefits for old age and the share of population aged 80 and over. From 2005 onwards, it also had a significant positive correlation with the self-perceived activity restrictions and the self-perceived health status as bad or very bad. This factor was therefore described as the alignment between old age related expenditure and elderly needs. The second PC was mainly negatively determined by the variables of LTC beds in institutions and recipients at home. It was therefore defined the factor of formal care, in terms of LTC beds in institutions and home recipients.

**Figure 2:**
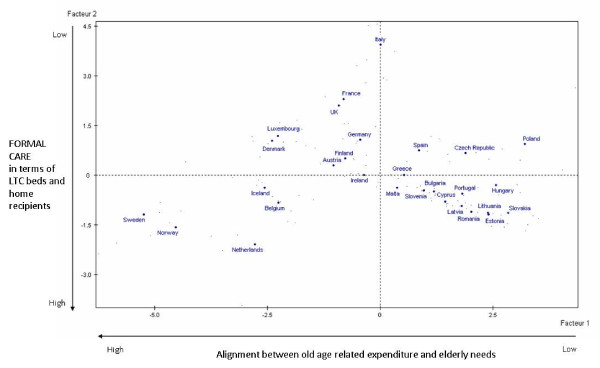
**MFA factorial space**.

Figure 2 represents the MFA factorial space. Comparing the average points of the countries, the first axis opposes Sweden, Norway and the Netherlands to Poland, Hungary and Slovakia, while there is an evident contraposition between Sweden, Norway and the Netherlands and Italy, the United Kingdom and France along the second axis. Combining the information expressed through these two factors, four main patterns of LTC appear, each of them being associated with some countries. Starting from the bottom left quadrant, there is a first group of countries - Nordic countries in particular (Sweden, Norway, the Netherlands, Iceland, Belgium) - especially characterized by high alignment between old age related expenditure and elderly needs, coupled with high formal care in terms of LTC beds and people cared for at home. In the top left quadrant there are countries characterized by high levels of both LTC and social benefits expenditures serving the elderly population, good health status and less restrictions on activities as self-perceived by the elderly, and a high share of population aged 80 and over (especially Luxembourg and Denmark). These countries also have a low level of formal care (especially Italy, France and the United Kingdom). In the top right part of the graph, Spain, the Czech Republic and Poland show a pattern of LTC characterized by lower LTC and social benefits expenditure, worse health status as self-perceived by the elderly, and a low level of formal care. Finally, in the bottom right quadrant, in countries as Slovakia, Hungary, Greece and others, the majority of south-eastern Europe, there is low alignment between old age related expenditure and elderly needs, though formal care is slightly above the mean.

### Trends

**Figure 3:**
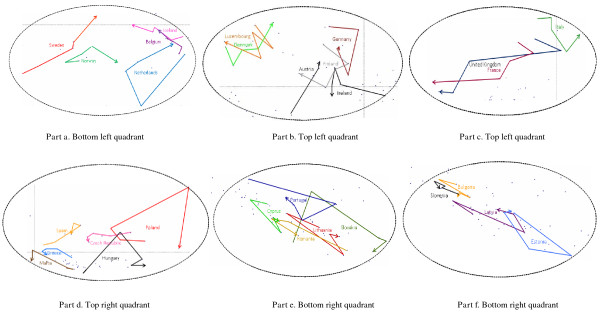
**Dynamic trends of the 29 European countries**. Detailed view of the four quadrants of the MFA factorial space.

Regarding the dynamic comparison (Figure 3), the arrows of Sweden, Iceland and Belgium in the bottom left quadrant, as well as those of Denmark and Austria in the top left quadrant, indicate a linear and clear dynamic. Sweden moves towards the top left quadrant, i.e., towards a reduction in the amount of formal care provided through beds and home care. Denmark and Austria follow a similar trend, while Iceland and Belgium show a slight reduction of formal care, but an increase in the alignment between public LTC and social benefits expenditure and elderly needs. Countries like Norway, the Netherlands, Germany and Finland have an overall stable dynamic along the axes. Ireland and Luxembourg have broken lines which denote unclear trends (Figure 3, *part a *and *part b*). The dynamic of the United Kingdom and France, still in the top left quadrant, is clearly towards an increase of the level of LTC and social benefits expenditure, of better self-perceived health and less self-perceived restrictions on activities and of increased level of formal care. Italy has a trend of reduction along the first factor, and an unclear trend along the second one (Figure 3, *part c*). The top right quadrant is characterized by the clear trend of the Czech Republic towards the negative semi axes of the first factor, by the circular movements of Poland and Hungary, and by unclear dynamic of the resting countries (Figure 3, *part d*). The bottom right quadrant is characterized by closed or irregular broken lines (e.g. Portugal, Lithuania, Estonia, Latvia), which denote either stable or irregular and unclear dynamics (Figure 3, *part e *and *part f*).

## Discussion

The main findings in this study are the different patterns of LTC that were identified. Countries characterized by closer alignment between old age related expenditure and elderly needs are those where the needs of the very old population seem to be properly met by the level of expenditure in the LTC sector and on social benefits. This pattern characterizes both Nordic and Western European countries. The majority of Nordic countries having higher levels of formal care are supply oriented systems, with a strong state responsibility for providing formal care [19]. This model of welfare also includes Denmark, where a varied range of adapted dwellings for older people have been developed in the last decades [20].

Western European countries mainly fulfil the needs of the elderly through social protection schemes based on cash for care, which are seen offering LTC services that are less expensive than traditional provision; these countries can be therefore defined as consumer choice oriented [21,22]. The cash programmes, both in tax funded and insurance based LTC systems, aim to give households choice over care decisions, fostering and supporting family care, developing care markets, and containing costs [22]. Three main types of programmes can be distinguished:

• personal budgets and consumer-directed employment of care assistants;

• payments to the person who needs care and can spend it as she/he likes, but has to acquire sufficient care;

• payments to informal caregivers as income support [1].

Also in some Nordic systems, cash for care schemes have been introduced to reform policies that were seen as too supply oriented, costly and unresponsive. This is the case of the *Personal Budget for Care and Nursing *in the Netherlands, the *Care Wage *in Norway, and the *Attendance Allowances *and the *Care Leave *in Sweden. These programs were all introduced to bring some flexibility into the LTC system. In Sweden, the employment of payments for care interventions, coupled with a reform policy that restricted care services to highly dependent elderly with limited family support, lowered the proportion of older people who received home care. This might explain the linear dynamic trend that emerged in the analysis [1,22].

The dynamic of both France and the United Kingdom is towards increasing resources addressed to the elderly and a surging amount of formal care. Since the mid 1990s France has undergone a process of reforming LTC, aiming to increase the number of recipients on the basis of a universal principle, and growing attention has been paid to elderly care after creating the "Plan for frail elderly people" in 2004 [23]. The United Kingdom has also faced some reforms of the LTC system in recent years, increasing the proportion of older dependent people who receive intensive home care packages [1].

Mediterranean and Central-South Eastern countries show less alignment between old age related expenditure and elderly needs. In addition, Mediterranean countries are especially characterized by lower levels of formal care, which is explained by the large amount of informal care which is mostly privately paid. In these countries, the source of welfare is traditionally the family, which provides the bulk of LTC, due to the way individuals perceive their responsibilities and the lack of other care options. In Greece, for example, relatives feel a duty to care which is reinforced by legal duty, social attitudes and lack of alternative care [24,25].

Other factors, such as the proportion of elderly people living alone, influence the availability of family care and the willingness of family members to provide it [26]. In terms of living arrangements, the proportion of old people living alone varies across Europe, with the lowest number still seen in some Southern European countries (19%) compared with 34%, 32% and 24% in the Nordic countries, Western and Eastern European nations respectively [27].

In Spain LTC was not defined as a specific service within health and social policy until recently [28]. Although several regions have begun specific programmes of building or subsidizing new facilities, there is a shortage of institutional care in many areas, and the majority of elderly people who receive care at home pay for private home help or rely on informal care [1]. The indistinct pattern of Italy and its irregular dynamic reflect the high variability and fragmented policy within the LTC system. This is due to two factors: the growing responsibility of regional governments in health care organization and funding, and demographic and cultural reasons. The supply of beds in LTC institutions as well as the employment of cash for care programmes, for example, differs substantially across Italian regions [23,28,29].

The linear dynamic of the Czech Republic is to a certain extent explained by a recent reform that laid new foundations for the provision and funding of social services and emphasized cash allowances paid to those in need of care. This marked a major turning point in the Czech LTC system, even though it remains to be seen what further developments the implementation of the new system will produce [30]. Central and South Eastern countries, despite the predominant role of informal care, show a level in the provision of formal care which is slightly above the average European level. Although the existing LTC infrastructure is very limited and poor, there is a lack of investment in new projects, and talks about privatization have not been supported by an adequate regulatory framework and financial support, these countries are service-oriented in the sense that residential care is the only alternative to informal arrangements and family networks [30]. In Hungary, social services for the elderly and the disabled do have a relatively well developed institutional network, however, they do not meet growing needs either in terms of number of places or quality of the services [1]. The unclear or irregular dynamics of these countries might be explained by the major transformations of their welfare systems in the past two decades, the majority oriented toward social insurance schemes. In Slovenia, for example, intensive debate resulted in a proposal for a LTC insurance scheme, but this scheme has yet to be implemented [30].

The scenario which arises from our analysis confirms the distinction between "weak family"/"individual" and "strong family" countries, which opposes the Northern and Western countries to Mediterranean and Southern ones. The latter ones are, in fact, characterized by traditional family structures, lower divorce rate, very late and increasing ages of leaving the parental home, most frequency of contact between parents and children [31].

These differences might result from religion traditions and cultural values, reflecting the European Protestant-Catholic dichotomy (Protestant emphasis on individualism *versus *Catholic family values) and the different social role attributed to men and women (high Femininity index in Nordic countries *versus *predominant Masculinity dimension of Latin ones) [32].

Our analysis has some limitations. First of all, the quality of the analysis is only as good as the quality and comparability of the international data allowed. For instance, data deriving from OECD are collected from national sources which vary from one country to another, so that variables such as Beds in institutions or Recipients in institutions may include different type of nursing homes or facilities. Also the variables expressing self-perceived health are influenced by subjectivity and international cultural differences, that could be the same that influence the self-reported level of "happiness", which is demonstrated to be higher in Nordic countries rather than in Southern ones [33].

Another limitation of the study is that some variables (i.e. the two related with the self- perceived health status and self-perceived activities limitation) were available only from 2004 onwards.

A relevant shortcoming of the MFA is that it fails to process missing elements, so that some relevant variables were excluded from the analysis [13]. In fact, international sources do not report systematic or complete data regarding, *inter alia*, the formal and informal workforce employed in the LTC sector and the share of elderly people living alone.

Still regarding the statistical method, different rules exist to retain factors that can lead to different results. However the explained variance criteria with a cut-off point of 60% was chosen in order to explain enough variance with as few meaningful factors as possible [34,35].

In addition, the interpretation of the factors generated in the MFA was "heuristic", meaning it was plausible and convenient even if not the only one possible; more than one interpretation can be made of the same data factored in the same way. Finally, the MFA does not identify causality.

The strengths of the study are many, however. It was an exploratory and dynamic analysis, that allowed us to take into consideration and to combine several important variables related to the LTC, thus having a global and integrated picture of them, allowing the confrontation of whole information, which is more rich than an examination parameter by parameter [15]. In addition, MFA has very good visualization properties, which makes it a suitable technique for data exploration [13].

Finally it was possible to perform the analysis despite the lack of two variables for the first year [12].

This study may represent a useful contribution to the resources for decision makers when dealing with the future common challenges that, apart from specific contexts and issues, all EU countries have to face. Building adequate systems of LTC is one of the most important challenges, which involves the integration and coordination of care between different service providers and between health and social care. The main critical issues are the organization of the LTC system and the balance between formal and informal care, residential care, home care and cash allowance programmes, and provision by the public and private sectors [27]. In the future, the availability of informal carers and their willingness to provide care will diminish, due to changes in family structure, growing participation of women in the labour market, and ageing of the partners and children who would otherwise supply informal care [2].

Given the general preference of elderly people to remain in their home for as long as possible with assistance, especially from their family, such informal care should be adequately supported by information, training, counseling, financial aid, employment leave and formalization of the role within the social security system [36].

Another relevant challenge will be the shortage of workers in this labour-intensive sector which requires adequate and well trained staff. Thus, policies aimed to improve the recruitment and the retention of qualified LTC staff and their working and contractual conditions will be needed [37]. Other critical points will be access to care and its quality, along with the fiscal sustainability, in terms of resources and expenditures allocated to the LTC sector and to the social protection systems [3]. It is recommended that countries should give priority to single entry point processes to manage LTC, in order to guarantee integrated and continuous care [33].

Finally, lack of international standard definitions, such as for disability and LTC expenditure, the use of different methodologies to gather data on the prevalence of old age disability and to measure the incidence of chronic conditions, some unclear demarcations (such as between the health care and social services sector, and between acute care and rehabilitation), and blurred boundaries between public and private sector provision, lead to the need for a more comparable, complete and up to date international database [7,30].

## Conclusions

In this study the issue of the LTC in Europe was analysed through the methodology of Multiple Factor Analysis, which is a feasible, rigorous and reproducible method that made it possible to combine several variables drawn from international databases. The application of this technique allowed us to take into consideration aspects concerning organizational settings, elderly needs and public LTC and social benefits expenditures over a number of years.

The analysis carried out, which is based on cross-sectional/time series and aggregated data, is an explorative and descriptive study. It represents an attempt to quantitatively reveal patterns and trends of LTC, allowing comparisons between the 29 European countries.

The relevant findings of the study are also a source of inspiration to stimulate further researches with lower aggregated data and in non-ecological design, that could overcome the limitations of this study and might reveal mechanisms behind these results useful to gain meaningful policy-making evidence.

## Abbreviations

LTC: Long Term Care; ADL: Activity of Daily Living; GDP: Gross Domestic Product; OECD: Organization for Economic Cooperation and Development; EU: European; MFA: Multiple Factor Analysis; PCA: Principal Component Analysis; PC: Principal Component.

## Competing interests

The authors declare that they have no competing interests.

## Authors' contributions

GD, and WR contributed to the conception of this paper. GD, LS, AA and VF conceived the study, AS, and AB provided data sources and participated in its design. GD and VF drafted the manuscript. LS, AA and DFI conceived the statistical methodology; LS, AA and VF provided the acquisition of data and performed statistical analyses. GD and AA had full access to all of the data in the study and take responsibility for the integrity of the data and the accuracy of the data analysis.

All authors read and approved the final manuscript.

## Pre-publication history

The pre-publication history for this paper can be accessed here:

http://www.biomedcentral.com/1472-6963/11/316/prepub

## Supplementary Material

Additional file 1 **Definition, source and indicator of the variables in the study**. Click here for file

## References

[B1] Organization for Economic Cooperation and Development Long term care for elderly people. Paris 2005 http://www.OECD.org

[B2] Commission of the European Communities The demographic future of Europe - from challenge to opportunity. Brussels 2006 http://europa.eu/legislation_summaries/employment_and_social_policy/situation_in_europe/c10160_en.htm

[B3] European Commission, Economic and Financial Affairs DG The 2009 Ageing Report: economic and budgetary projections for the EU-27 Member States (2008-2060). Luxembourg 2009 http://ec.europa.eu/economy_finance/publications/publication_summary14911_en.htm

[B4] Lafortune G Balestat G Trends in severe disability among elderly people: assessing the evidence in 12 OECD countries and the future implications [Organization for Economic Cooperation and Development, Health Working Paper no. 26]. Paris 2007 http://www.oecd.org/document/25/0,3343,en_2649_33929_2380441_1_1_1_1,00.html

[B5] Hoxley H Policies for healthy ageing: an overview [Organization for Economic Cooperation and Development, Health Working Paper no. 42]. Paris 2009 http://www.oecd.org/document/25/0,3343,en_2649_33929_2380441_1_1_1_1,00.html

[B6] Oliveira Martins J Projecting OECD health and long term care expenditures: What are the main drivers? [Organization for Economic Cooperation and Development. Economics Department Working Paper no. 477]. Paris 2006 http://www.oecd.org/LongAbstract/0,3425,en_2649_34113_36085941_1_1_1_1,00.html

[B7] European Commission, Social Affairs and Equal Opportunities DG Long Term Care in the European Union 2008 http://ec.europa.eu/employment_social/news/2008/apr/long_term_care_en.pdf

[B8] OECD Health Data 2009 http://www.OECD.org

[B9] Eurostat Statistics http://epp.eurostat.ec.europa.eu/portal/page/portal/eurostat/home/

[B10] Escofier B Pagès J Multiple factor analysis Computational Statistics & Data Analysis 1990 18 121 14022085452

[B11] Petigas P Poulard JC A multivariate indicator to monitor changes in spatial patterns of age-structured fish populations Aquat Living Resour 2009 22 165 171 10.1051/alr/2009018

[B12] Abdi H Valentin D Salkind N Multiple Factor Analysis (MFA) Encyclopedia of measurement and statistics 2007 1 14

[B13] Stanimirova I Walczak B Massart DL Multiple factor analysis in environmental chemistry Analytica Chimica Acta 2005 545 1 12 10.1016/j.aca.2005.04.054

[B14] Mazouni N Gaertner JC Deslous Papli JM Composition of biofouling communities on suspended oyster coltures: an in situ study on interaction with the water column Marine Ecology Progress Series 2001 214 93 102

[B15] Wong S Gauvrit H Cheaib N Carré F Carrault G Multiple factor analysis as a tool for studying the effect of physical training on the autonomic nervous system Computers in Cardiology 2002 29 437 440

[B16] Ainleya DG Speara LB Tynanb CT Barthc JA Piercec SD Fordd RG Cowlesc TJ Physical and biological variables affecting seabird distributions during the upwelling season of the northern California Current Deep-Sea Research 2005 52 123 143

[B17] Petitgas P Poulard JC Multi-factorial Analysis (MFA) and Spatial Indicators 2004 Fisboat: fisheries independent survey-based operational assessment tools. Nantes 129 132

[B18] Porcu R Servizi sanitari, popolazione e mobilità sanitaria della Sardegna: un'analisi multiway. *(Health care services, population and health care mobility in Sardinia: a multiway analysis)* Difesa Sociale LXXXVI 2007 3 57 74

[B19] American Association for Retired Persons Public Policy Institute European experience with Long Term Care. France, the Netherlands, Norway and the United Kingdom 2006 http://www.aarp.org/research/international/report/leadershipstudyreports.html

[B20] Schulz E The long term care system in Denmark. [European Network of Economic Policy Research Institute, Research Report no. 73] 2010 DIW Berlin (Deutsches Institut fuer Wirtschaftforschung http://www.ancien-longtermcare.eu/node/27

[B21] Fernandez JL Forder J Trukeschits B Rokosaova M McDaid D How can European states design efficient, equitable and sustainable funding systems for long term care for older people? 2009 World Health Organization, Regional Office for Europe and European Observatory on Health Systems and Policies

[B22] Da Roit B Le Bihan B Similar and yet so different: cash-for-care in six European countries' long-term care policies The Milbank Quarterly 2010 88 3 286 309 10.1111/j.1468-0009.2010.00601.x 20860573 PMC3000929

[B23] Da Roit B Le Bihan B Österle A Long-term care policies in Italy, Austria and France: variations in cash-for-care schemes Social policy and administration 2007 41 653 671 10.1111/j.1467-9515.2007.00577.x

[B24] Bolin K Lindgren B Lundborg P Informal and formal care among single living elderly in Europe Health Economics 2008 17 3 393 409 10.1002/hec.1275 17768700

[B25] Gibson MJ Gregory SR Pandya SM Long Term Care in developed nations: a brief overview 2003 AARP Public Policy Institute http://www.uncioa.org/agelib/record.asp?RecordID=3035

[B26] Garcés J Ròdenas F Sanjosé V Towards a new welfare state: the social sustainability principle and health care strategies Health Policy 2003 65 201 215 10.1016/S0168-8510(02)00200-2 12941489

[B27] Rechel B Doyle Y Grundy E McKee M How can health systems respond to population ageing? World Health Organization, Regional Office for Europe and European Observatory on Health Systems and Policies 2009

[B28] Costa-Font J Devolution, diversity and welfare reform: Long-term care in the 'Latin Rim' Social Policy Administration 2010 44 4 481 494 10.1111/j.1467-9515.2010.00724.x

[B29] Damiani G Colosimo S Sicuro L Burgio A Battisti A Solipaca A Baldassarre G Crialesi R Milan G Tamburrano T Ricciardi W An ecological study on the relationship between supply of beds in long-term care institutions in Italy and potential care needs for the elderly BMC Health Services Research 2009 9 174 186 10.1186/1472-6963-9-174 19778449 PMC2762968

[B30] Österle A Long-term Care in Central and South-Eastern Europe: challenges and perspectives in addressing a 'new' Social Risk Social Policy & Administration 2010 44 4 461 480 10.1111/j.1467-9515.2010.00723.x22102793

[B31] Axel Börsch-Supan et al First Results from the Survey of Health, Ageing and Retirement 2008 Mannheim Research Institute for the Economics of Aging (MEA)

[B32] Hofstede G Culture's consequences: comparing values, behaviors, institutions, and organizations across nations 2001 2 Thousand Oaks, CA: Sage

[B33] Veenhoven R Diener E, Suh EM Freedom and happiness. A comparative study in 46 nations in early 1990s 2000 'Culture and subjective wellbeing' MIT press, Cambridge, MA USA

[B34] Dunteman GH Principal Component Analysis 1989 SAGE Publications

[B35] Suhr DD Principal Component Analysis vs. Exploratory Factor Analysis, Paper 203-30

[B36] Burwell B Jackson B The disabled elderly and their use of long-term care 1994 http://www.aspe.hhs.gov

[B37] Colombo F The long term care workforce: overview and strategies to adapt supply to a growing demand [Organization for Economic Cooperation and Development, Health Working Papers no.44]. Paris 2009 http://www.oecd.org/LongAbstract/0,3425,en_2649_34113_36085941_1_1_1_1,00.html

