# Supplementary material for: Patterns of Long Term Care in 29 European countries: evidence from an exploratory study
Source: BMC Health Serv Res. 2011 Nov 18;11:316. doi: 10.1186/1472-6963-11-316 (PMC3228675; doi:10.1186/1472-6963-11-316)
Supplement: Additional file 1 — Definition, source and indicator of the variables in the study. [file 1472-6963-11-316-S1.DOC]

**Additional file** 1

| **Variable** | **Definition** | **Institutional source** | **Specific source** | **Indicator** |
| --- | --- | --- | --- | --- |
| LTC BEDS IN INSTITUTIONS | Beds in all types of nursing and residential care facilities dedicated to long-term nursing care and beds for palliative care in all types of nursing and residential care facilities. | OECD Health Data 2009 | National sources | Rate per 100 population aged 65 and over |
| LTC RECIPIENTS IN INSTITUTIONS | People receiving formal (paid) LTC in institutions (other than hospitals). The services received can be publicly or privately financed. Institutions refer to nursing and residential care facilities which provide accommodation and LTC as a package. They include specially designed institutions or hospital-like settings where the predominant service component is LTC and the services are provided for people with moderate to severe functional restrictions. | OECD Health Data 2009 | National sources | Rate per 100 population aged 65 and over |
| LTC RECIPIENTS AT HOME | People receiving formal (paid) LTC at home. The services can be publicly or privately financed. LTC at home is provided to people with functional restrictions who mainly reside at their own home. It also applies to the use of institutions on a temporary basis to support continued living at home - such as in the case of community care and day care centres and in the case of respite care. Home care also includes specially designed or adapted living arrangements for persons who require help on a regular basis while guaranteeing a high degree of autonomy and self-control. | OECD Health Data 2009 | National sources | Rate per 100 population aged 65 and over |
| SELF-PERCEIVED HEALTH AS BAD OR VERY BAD, PEOPLE AGED 65 | Auto-evaluation of the general health state (i.e. any temporary health problem is not considered) by respondents. We took into consideration categories “ bad” and “very bad” in people aged 65 and over. | Eurostat Statistics | European Statistic and Living Condition (EU-SILC) survey, launched in 2004 | Rate per 100 population aged 65 and over |
| SELF- PERCEIVED LIMITATIONS IN DAILY ACTIVITIES (ACTIVITY RESTRICTIONS FOR AT LEAST THE LAST SIX MONTHS), PEOPLE AGED 65+ | Auto-evaluation by the respondents of the extent of which they are limited in activities people usually do because of health problems for at least the last six months. | Eurostat Statistics | European Statistic and Living Condition (EU-SILC) survey, launched in 2004 | Rate per 100 population aged 65 and over |
| TOTAL LTC EXPENDITURE(HC.3+HC.R.6) | It includes “health” (HC.3) and “social” ( HC.R.6) components of LTC. HC.3 refers to “Services of long term nursing care”: it is the medical component of LTC. It comprises ongoing health and nursing care given to in-patients who need assistance on a continuing basis due to chronic impairments and a reduced degree of independence and activities of daily living (ADLs). The main personal care component is frequently provided in combination with help with basic medical services such as wound dressing, medication, health monitoring and with lower level care of home help or help with the IADLs. In-patient LTC is provided in institutions or community facilities.  HC.R.6 refers to “ Administration and provision of social services in kind to assist living with diseases and impairment”. This item comprises (non-medical) social services in kind provided to persons with health problems and functional limitations or impairments where the primary goal is the social and vocational rehabilitation or integration. Social services of LTC comprises home help and assistance predominantly aimed to help with IADLs restrictions. | Eurostat Statistics | Database based on a co-operation between Eurostat, OECD and WHO. Data on expenditure are largely based on surveys and administrative (register) data sources in the countries. The main source of definitions and classifications of expenditure is the System of Health Account (SHA) | Rate per 100 population aged 65 and over (in million euros) |
| SOCIAL PROTECTION BENEFITS OLD AGE | The provision of social protection against the risks linked to old age: loss of income, inadequate income, lack of independence in carrying out daily tasks, reduced participation in social life, and so on. Medical care of the elderly is not taken into account.  Benefits for the old age function include (1) cash benefits, such as old age pensions, anticipated old age pensions, partial retirement pension, care allowance and other cash benefits, and (2) benefits in kind, such as accommodation, assistance in carrying out daily tasks, other benefits in kind. | Eurostat Statistics | ESSPROS (European System of integrated Social PROtection Statistics, which is an instrument of statistical observation that enables international comparisons on the administrative national data on social protection in the EU Member States. | Rate per 100 population aged 65 and over (in million euros) |
| POPULATION  OVER 80 |  | Eurostat Statistics |  | Rate per 100 total population |
